# Updated study on demographic and ocular biometric characteristics of cataract patients indicates new trends in cataract surgery

**DOI:** 10.1038/s41598-025-02311-5

**Published:** 2025-05-19

**Authors:** Jiancheng Mu, Feng Xu, Wanyue Guo, Chuhuan Sun, Bosen Peng, Qing Huang, Wei Fan

**Affiliations:** Department of Ophthalmology, West China Hospital, Sichuan University, Chengdu, China

**Keywords:** Biometry, Cataract, Demography, Myopia, Ocular, Refractive surgery, Diseases, Health care, Medical research

## Abstract

To assess changes in the demographics and ocular biometric characteristics of cataract patients over the past decade to identify trends that may impact cataract surgery as well as design and selection of intraocular lenses. Retrospective study. Clinicodemographic and ocular biometric data from swept-source optical coherence tomography were retrospectively analyzed for 25,192 eyes of 25,192 patients aged 40 and older who underwent cataract surgery between 2020 and 2023 at West China Hospital of Sichuan University. As patient age increased, axial length, anterior chamber depth, aqueous depth, and white-to-white tended to decrease, whereas corneal refractive power and lens thickness tended to increase. Among all patients, 19.66% had axial lengths exceeding 26 mm, and 1.72% had a history of refractive surgery; the incidence of such surgery increased significantly from 2020 to 2023. Compared to patients without a history of refractive surgery, those with such a history were 12 years younger and larger proportions had axial lengths exceeding 26 mm or corneal refractive power below 38 D. The frequencies of high myopia and of previous refractive surgery among cataract patients increased between the 2010s and the 2020s, and the age of cataract patients at the time of surgery fell during that interval. These changes in ocular and demographic characteristics may necessitate updates in preparations before cataract surgery, procedures during surgery, management after surgery, as well as design and selection of intraocular lenses.

**Supplementary Information:**

The online version contains supplementary material available at 10.1038/s41598-025-02311-5.

## Introduction

Cataracts, the leading cause of blindness, are a major public health concern worldwide^1^. The condition, in which the lens clouds over, affects primarily older adults, though it can occur at any age as a result of genetics, trauma, or and/or disease^2^. As the global population ages, the number of individuals affected by cataracts is expected to rise. In parallel, the incidences of myopia and of refractive surgeries to correct it are also rising^3–5^. As a result, growing proportions of individuals undergoing cataract surgery have a history of high myopia and refractive surgery^6^. Some patients may have even undergone two different refractive surgeries in one eye^7^.

Cataract surgeons must take these factors into account because refractive surgery alters ocular morphology^8^, which can complicate power calculations for selecting an intraocular lens^9^. Fortunately, the formulas for power calculations can be improved to take into account previous refractive surgeries^10–13^. Thus, clinicians should be aware of the ocular and demographic characteristics of cataract patients in order to optimize their treatment and postoperative management. In previous work, our group analyzed such characteristics in a cohort of individuals at least 50 years old who underwent cataract surgery between November 2011 and August 2014 at our hospital, one of the largest comprehensive hospitals in China^14^. The rapidly changing characteristics of such patients prompted us to repeat the analysis in a cohort of individuals at least 40 years old who underwent cataract surgery at the same hospital between 2020 and 2023. Comparing the two cohorts a decade apart may help predict how cataract surgery and intraocular lens selection need to adapt for the future.

## Results

### Demographic characteristics of cataract surgery patients in Southwest China

**Fig. 1:**
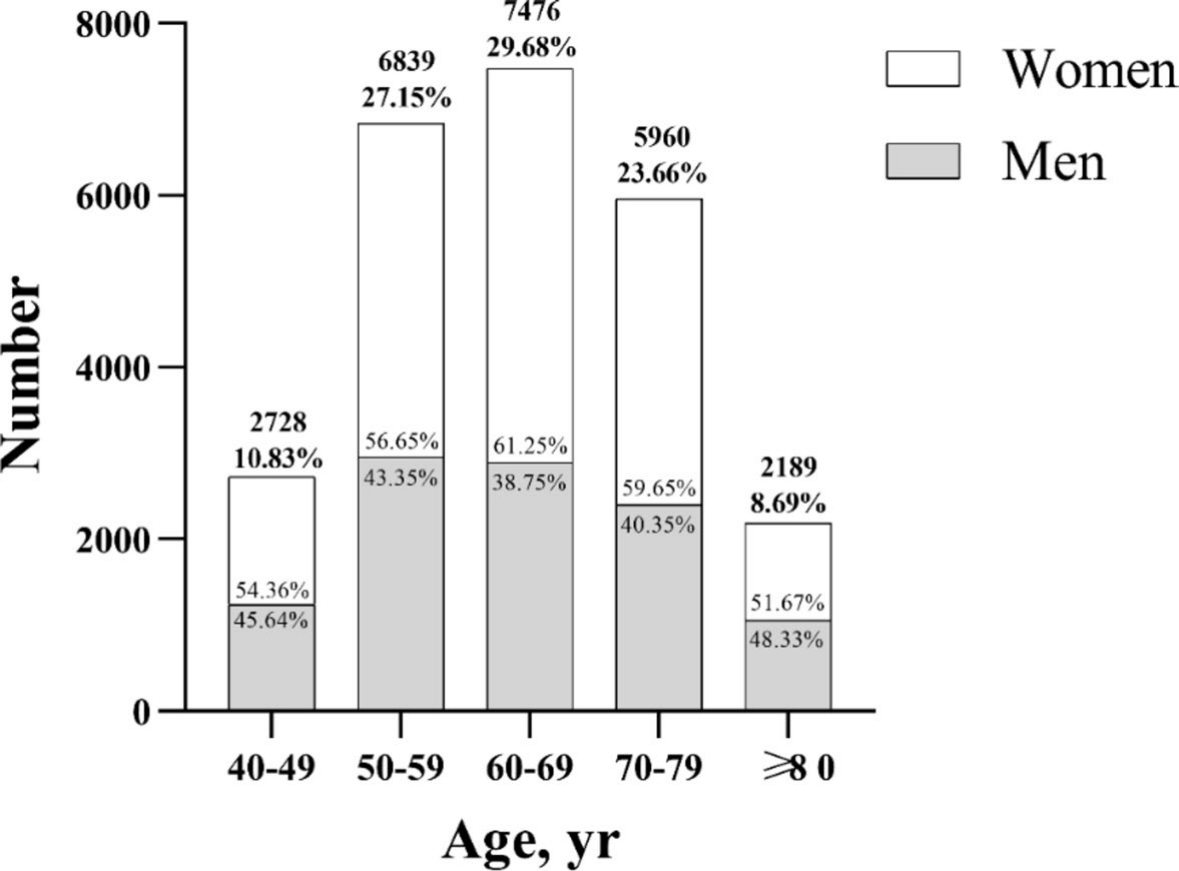
Distribution of numbers and sex of individuals across different age ranges in the cohort.

We analyzed 25,192 eyes from the same number of cataract patients who underwent surgery at our hospital between January 2020 and December 2023 (Table 1). Slightly more than half the patients were women (58.04%) (Fig. 1). The overall average age of patients was 63.84 ± 11.23 years (range, 40 to 103 years), while the average age among patients aged 50 years and older was 66.09 ± 9.67 years (Supplementary Table 1). Nearly equal proportions of the cohort were 50–59 years old (27.15%) or 60–69 years old (29.68%), a slightly smaller proportion was 70–79 years old (23.66%), and much smaller proportions were 40–49 years old (10.83%) or at least 80 years old (8.69%).

**Table 1:** Numbers of cataract cases in our cohort, stratified by year and history of refractive surgery.

Group	Year				Total
	2020	2021	2022	2023	
All	3738 (14.84)	6363 (25.26)	6735 (26.73)	8356 (33.17)	25,192 (100)
Previous refractive surgery					
No	3689 (98.69)	6269 (98.52)	6610 (98.14)	8190 (98.01)	24,758 (98.28)
Yes	49 (1.31)	94 (1.48)	125 (1.86)	166 (1.99)	434 (1.72)

Values are n (%).

Of the 25,192 patients, 434 (1.72%) had a history of refractive surgery, and the prevalence of such history increased significantly from 2020 to 2023 (*P* < 0.01; Table 1). Of the 434 patients with such history, 10 (2.30%) had undergone two refractive surgeries in one eye (Supplementary Table 2). The most frequent refractive surgery was laser-assisted in situ keratomileusis (LASIK) (196 of 424, 46.23%), followed by photorefractive keratectomy (PRK) (134, 31.60%), radial keratotomy (RK) (81, 19.10%), and collamer lens implantation (ICL) (3, 0.71%).

**Fig. 2:**
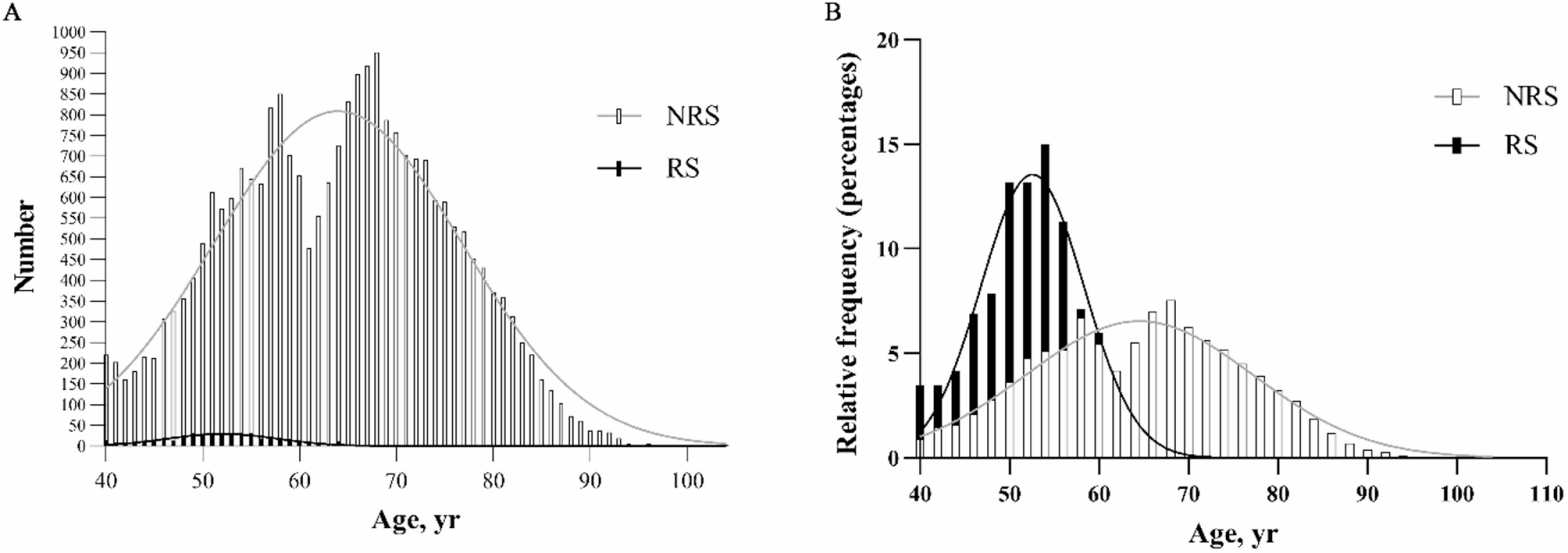
Age distribution of patients with a history of refractive surgery (RS) or not (NRS), in terms of (**A**) absolute numbers and (**B**) percentages. The solid lines represent Gaussian modeling.

Compared to patients without a history of refractive surgery, those with such a history were significantly more likely to be women (67.97 vs. 57.87%, *P* < 0.001) and they were significantly younger (52.18 ± 6.19 vs. 64.04 ± 11.19 years, *P* < 0.0001) (Table 2). Women predominated over men regardless of history of refractive surgery. Patients without a history of refractive surgery spanned the entire age range from 40 to 90 years, whereas those with such a history concentrated nearly entirely between 40 and 60 years old (397 of 434, 91.47%), accounting for only 3.88% of the 10,229 cataract patients in this age group (Fig. 2).

### Ocular biometric characteristics of cataract surgery patients in Southwest China

Across all patients, axial length (AL) was 24.50 ± 2.60 mm and showed positive skew of 1.5674 with kurtosis of 2.7572 (Supplementary Fig. 1A). Mean simulated keratometry (MK) was 43.96 ± 1.85 D and showed negative skew of -0.8863 with kurtosis of 5.788 (Supplementary Fig. 1B). Mean total keratometry (MTK) was 43.94 ± 1.88 D and mean anterior keratometry (MAK) was 48.97 ± 2.06 D, and both parameters showed negative skew with respective kurtosis values of 6.336 and 5.788 (Supplementary Figs. 1G-H). Mean posterior K (MPK) was − 5.92 ± 0.26 D and it showed positive skew of 0.3320 with kurtosis of 5.162 (Supplementary Fig. 1I). Corneal astigmatism (CA) was 0.95 ± 0.85 D and showed a strongly positive skew of 5.2818 with kurtosis of 63.0628 (Supplementary Fig. 1C). Anterior chamber depth (ACD) was 3.04 ± 0.50 mm and aqueous depth (AQD) was 2.50 ± 0.50 mm, and both parameters showed slightly positive skew with respective kurtosis values of 1.7655 and 1.7163 (Supplementary Figs. 1D-E). Lens thickness (LT) was 4.45 ± 0.52 mm and showed mildly negative skew of -0.3163 with kurtosis of 8.5143 (Supplementary Fig. 1F). Central corneal thickness (CCT) was 0.5385 ± 0.0411 mm and mean horizontal corneal diameter (“white-to-white”, WTW) was 11.66 ± 0.46 mm, and both parameters showed positive skew with respective kurtosis values of 13.3072 and 4.3944 (Supplementary Figs. 1 J-K).

With increasing age, nearly all ocular biometric parameters tended to decrease continuously (Supplementary Fig. 2). Exceptions were MK and LT, which increased with age; CA and MPK, which initially decreased and then increased with age; and CCT, which did not vary much across the age range. CA decreased from 1.05 ± 0.90 D among those 40–49 years old to 0.83 ± 0.80 D among those 60–69 years old, after which it rose gradually to 1.11 ± 0.86 D among those at least 80 years old (Supplementary Table 1).

Just over half the patients with a history of refractive surgery (228 of 434, 52.53%) had MK smaller than 38 D (Supplementary Table 3). Compared to patients without a history of refractive surgery, those with such a history showed higher AL, ACD, AQD; lower MK, MTK, MAK, LT and CCT; and similar CA, MPK and WTW (Table 2 and Supplementary Fig. 3). Subgroups of patients who underwent RK, PRK or LASIK showed significantly longer AL, ACD, AQD and WTW, but significantly smaller MK, MTK, MAK, absolute MPK values and LT, than patients without a history of refractive surgery (Supplementary Fig. 4). Among these three subgroups by type of refractive surgery, CA and MPK were significantly bigger, CCT significantly thicker, and A/P significantly smaller in those who underwent radial keratotomy than in the other two subgroups. Conversely, A/P was significantly larger in those who had a history of photorefractive keratectomy or LASIK than in those who had no history of refractive surgery. CCT was significantly thicker in those with a history of radial keratotomy, but significantly thinner in those with a history of PRK or LASIK surgery, than in those with no history of refractive surgery. Subgroups who had a history of other types of refractive surgery were too small to be analyzed.

**Table 2:** Demographic and ocular biometric characteristics of our cohort, stratified by type of previous refractive surgery.

Characteristic	No surgery	Any surgery type	Type of refractive surgery							*P*
			RK	PRK	LASEK	LASIK	Collamer lens	RK + PRK	RK + LASIK	
n (% of 25,192)	24,758 (98.28)	434 (1.72)	81 (0.32)	134 (0.53)	10 (0.04)	196 (0.78)	3 (0.01)	4 (0.02)	6 (0.02)	
Age, yr	64.04 ± 11.19	52.18 ± 6.19	54.27 ± 5.61	53.77 ± 5.75	47.50 ± 5.52	50.36 ± 6.12	47.00 ± 7.55	57.00 ± 5.42	54.83 ± 4.88	< 0.0001
Sex										
Men	10,431 (42.13)	139 (32.03)	32 (39.51)	38 (28.36)	1 (10)	63 (32.14)	2 (66.67)	1 (25.00)	2 (33.33)	
Women	14,327 (57.87)	295 (67.97)	49 (60.49)	96 (71.64)	9 (90)	133 (67.86)	1 (33.33)	3 (75.00)	4 (66.67)	
AL (mm)	24.42 ± 2.55	28.56 ± 2.44	28.43 ± 2.33	28.89 ± 2.47	28.44 ± 2.83	28.44 ± 2.40	31.65 ± 1.69	26.98 ± 1.63	26.85 ± 2.89	< 0.0001
MK (D)	44.07 ± 1.63	37.74 ± 2.54	36.51 ± 3.09	37.85 ± 2.33	38.57 ± 2.81	38.02 ± 2.27	41.54 ± 2.19	38.24 ± 1.63	39.56 ± 1.67	< 0.0001
CA (D)	0.95 ± 0.85	1.04 ± 0.92	1.50 ± 1.08	1.06 ± 1.06	0.94 ± 0.51	0.87 ± 0.69	0.99 ± 1.14	0.54 ± 0.67	0.93 ± 0.52	0.0437
ACD (mm)	3.03 ± 0.50	3.33 ± 0.38	3.36 ± 0.55	3.32 ± 0.35	3.28 ± 0.38	3.35 ± 0.32	3.21 ± 0.21	3.02 ± 0.23	3.08 ± 0.38	< 0.0001
AQD (mm)	2.49 ± 0.50	2.84 ± 0.39	2.80 ± 0.56	2.84 ± 0.35	2.80 ± 0.34	2.88 ± 0.32	2.60 ± 0.22	2.52 ± 0.22	2.58 ± 0.41	< 0.0001
LT (mm)	4.46 ± 0.52	4.28 ± 0.35	4.32 ± 0.36	4.34 ± 0.34	4.13 ± 0.37	4.22 ± 0.34	4.27 ± 0.18	4.53 ± 0.19	4.44 ± 0.35	< 0.0001
MTK (D)	44.07 ± 1.61	37.27 ± 2.61	36.36 ± 3.22	37.25 ± 2.36	37.79 ± 3.52	37.51 ± 2.36	41.58 ± 2.04	38.13 ± 1.48	39.44 ± 1.78	< 0.0001
MAK (D)	49.09 ± 1.82	42.04 ± 2.82	40.67 ± 3.44	42.17 ± 2.59	42.97 ± 3.14	42.35 ± 2.53	46.28 ± 2.43	42.61 ± 1.81	44.07 ± 1.86	< 0.0001
MPK (D)	−5.92 ± 0.25	−5.56 ± 0.52	−4.82 ± 0.60	−5.68 ± 0.34	−5.84 ± 0.22	−5.76 ± 0.29	−5.55 ± 0.44	−5.84 ± 0.23	−5.73 ± 0.31	< 0.0001
A/P	1.13 ± 0.02	1.12 ± 0.09	1.12 ± 0.09	1.27 ± 0.08	1.28 ± 0.10	1.28 ± 0.07	1.13 ± 0.03	1.27 ± 0.01	1.21 ± 0.05	0.0211
CCT (mm)	0.5393 ± 0.0403	0.4937 ± 0.0579	0.5615 ± 0.0457	0.4796 ± 0.0482	0.4853 ± 0.0991	0.4735 ± 0.0418	0.6009 ± 0.0378	0.4988 ± 0.0427	0.5043 ± 0.0627	< 0.0001
WTW (mm)	11.66 ± 0.46	11.82 ± 0.39	11.88 ± 0.33	11.80 ± 0.39	11.86 ± 0.32	11.81 ± 0.40	11.63 ± 0.72	11.72 ± 0.36	11.55 ± 0.63	< 0.0001

Values are n (%) or mean ± SD. *P*: Differences between groups with and without a history of refractive surgery.

*LASEK* laser assisted sub-epithelial keratectomy, *LASIK* laser in-situ keratomileusis, *PRK* photorefractive keratectomy, *RK* radial keratotomy, *ACD* anterior chamber depth, *AL* axial length, *A/P* ratio of anterior to posterior corneal surfaces, *AQD* aqueous depth, *CA* corneal astigmatism, *CCT* central corneal thickness, *LT* lens thickness, *MAK* mean anterior keratometry, *MK* mean simulated keratometry, *MPK* mean posterior keratometry, *MTK* mean total keratometry, *WTW* white-to-white.

### High myopia

**Fig. 3:**
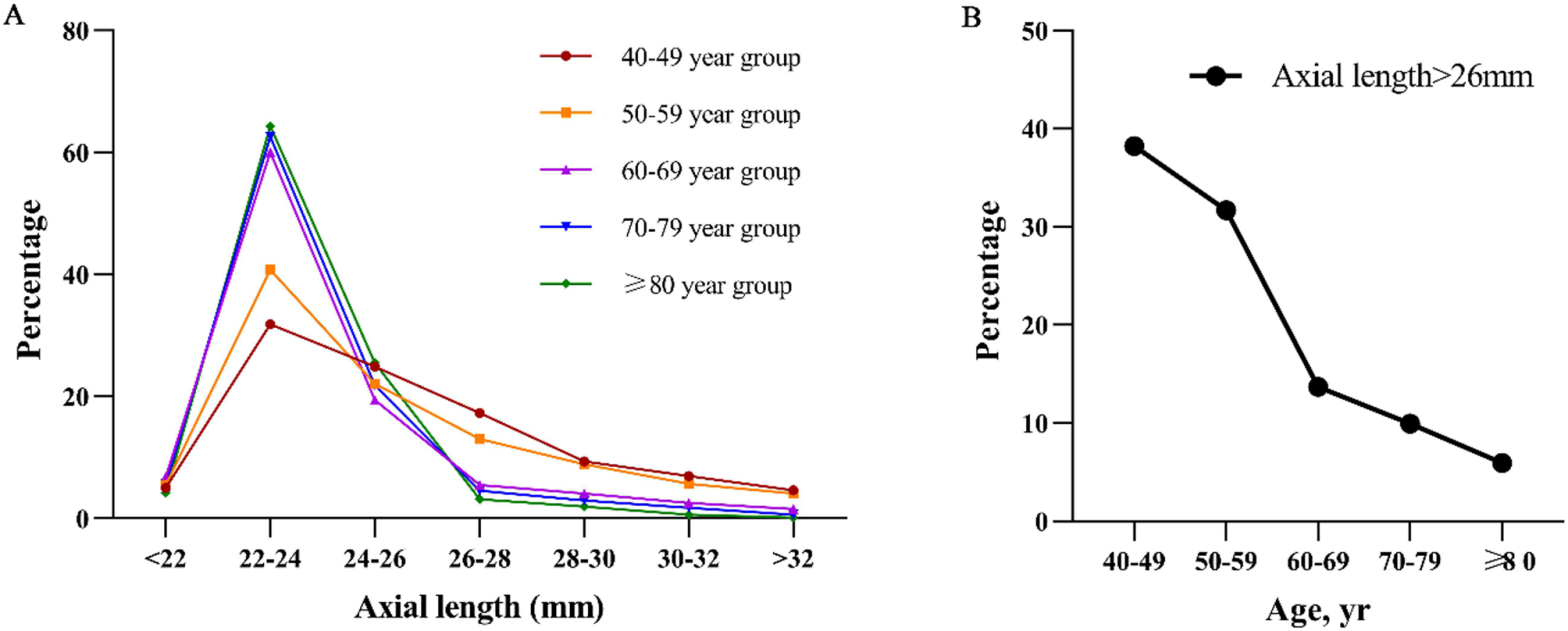
Axial length and age in our cohort. (**A**) Prevalence of different ranges of axial length in different age groups. (**B**) Prevalence of axial length > 26 mm in different age groups.

The prevalence of high myopia, which we defined as AL > 26 mm, was 19.66% (4,954 of 25,192) across the entire population, and the condition was particularly prevalent among those 40–49 years old (38.23%) and 50–59 years old (31.67%) (Supplementary Table 4), with prevalence tending to decrease with increasing age (Fig. 3). The prevalence of AL > 26.5 mm was 17.15% (4,320 of 25,192).

Prevalence was much higher among those with a history of refractive surgery (84.79%, 368 of 434) than among those without such history (18.52%, 4,586 of 24,758), such that patients with a history of refractive surgery accounted for 7.43% (368) of the 4,954 cataract patients with high myopia (Supplementary Table 3). In fact, just over half of patients with a history of refractive surgery (54.61%, 237 of 434) had AL > 28 mm. Such long AL was much less prevalent among those without a history of refractive surgery (10.52%, 2,605 of 24,758) and among all patients in the cohort (11.28%, 2,841 of 25,192) (Supplementary Table 3). Similarly, prevalence of AL > 30 mm was much higher among those with a history of refractive surgery (29.72%, 129 of 434) than among those without such history (5.35%, 1,324 of 24,758; Supplementary Table 3).

### Corneal astigmatism

**Fig. 4:**
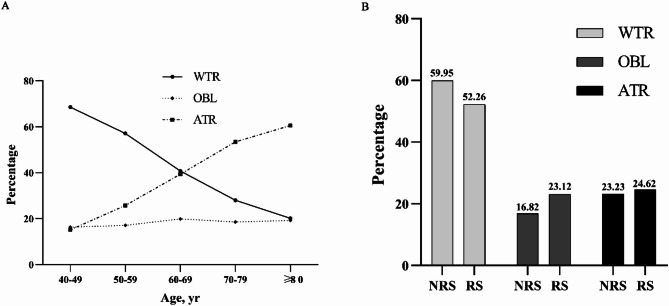
Corneal astigmatism and age in our cohort. (**A**) Prevalence of “with-the-rule” (WTR), “against-the-rule” (ATR) and “oblique” (OBL) astigmatism in different age groups. (**B**) Comparison of the three types of astigmatism between patients aged 40 to 60 with a history of refractive surgery (RS) and those in the same age range without such a history (NRS).

In the entire 2020–2023 cohort, the prevalence of astigmatism was 51.48% (12,970 of 25,192) in the case of > 0.75 D, 35.21% (8,869) in the case of > 1 D, and 8.06% (2,030) in the case of > 2 D (Supplementary Table 3). The prevalence of astigmatism > 0.75 D was higher among patients with a history of refractive surgery (60.14%, 261 of 434) than among those without such history (51.33%, 12,709 of 24,758). As age increased, so did the prevalence of against-the-rule astigmatism (ATR), while the prevalence of with-the-rule astigmatism (WTR) decreased (Fig. 4A). Among patients aged 40 to 60, WTR astigmatism was less prevalent, whereas ATR and oblique astigmatism (OBL) were more prevalent in those with a history of refractive surgery compared to those without such a history (Fig. 4B).

## Discussion

This analysis of a large population of cataract patients in southwest China updates and extends a similar analysis that we conducted on a patient cohort treated at the same medical center between late 2011 and mid-2014^14^. The medical center is one of the largest in China and draws patients from Sichuan, the most populous province in the western part of the country, as well as neighboring provinces. Thus, the results from these two studies provide a comprehensive understanding of the demographic and ocular characteristics of cataract patients who may be representative of patients in many parts of the country.

Our analyses suggest that over the past decade, the prevalence of previous refractive surgery has increased among cataract patients. The proportion of cataract patients in southwest China with a history of refractive surgery increased from 1.31% in 2020 to 1.99% in 2023. Consistent with these results, the proportion of cataract patients in a pan-European study who had a history of refractive surgery increased steadily from 0.06% in 2008 to 0.22% in 2013^15^. The eyes in our cohort that underwent refractive surgery differed significantly from those that never underwent refractive surgery: they had longer AL, showed smaller K, more irregular astigmatism, and thinner CCT, which challenge the accuracy of biometric measurement and calculation of IOL power. Ophthalmologists and cataract surgeons should be aware of these demographic and ocular changes, and they should update accordingly how they advise, treat and manage cataract patients, including how they manage younger patients’ expectations about the quality of their vision and the independence of glasses. Clinicians should also take these changes into account when selecting an intraocular lens.

Our analyses of Chinese cohorts in the 2010s and 2020s, as well as several previous studies in the USA, the UK, and New Zealand^16–19^, indicate higher prevalence of women than men among cataract patients, regardless of history of refractive surgery. This may reflect greater concern among women about aesthetics or quality of life, or lower tolerance of contact lenses. Future studies should clarify the reasons for this apparent sex bias.

Our analyses suggest that over the past decade, cataract patients have been getting younger. The average age of cataract patients in our cohort from the 2020s, 63.84 ± 11.23 year, was smaller than that in our cohort from the 2010s, 68.58 ± 9.07 year^14^. Considering that the previous cohort included only patients aged 50 and above, we then analyzed cataract patients aged 50 years and older in the cohort from the 2020s and showed the average age, 66.09 ± 9.67 year, was still smaller than that in our cohort from the 2010s. In contrast, the average age in a European cohort from the 2010s was 74^15^, higher than the age of our Chinese cohort in the 2010s. The difference in cataract surgery age between Europe and China can be attributed to variations in healthcare accessibility, lifestyle demands, cataract progression rates, and support systems for the elderly. These findings may reflect a growing emphasis on improving vision and quality of life through surgical advances. In our newer Chinese cohorts, cataract patients with a history of refractive surgery were more than a decade younger than those without such a history, and the same was true in the European cohort from the 2010s^15^. Those who have undergone refractive surgery tend to have higher expectations of vision quality and lower tolerance for vision decline caused by cataracts, so they tend to opt for cataract surgery as soon as their vision is affected, often at an earlier age than those who have not undergone refractive surgery^15^. Indeed, the fact that most of our 2020–2023 cohort was 40–60 years old may reflect that the individuals who took advantage of radial keratotomy after its introduction in China in the 1980s^20^ or excimer and femtosecond laser corneal refractive surgery after its introduction in the 1990s^21^ have reached ages at which cataracts tend to develop.

Our analyses suggest that over the past decade, the prevalence of high myopia has increased among cataract patients. The prevalence of highly myopic eyes in southwest China has increased from the 2010s to today. The proportion of eyes with AL > 26.5 mm was much higher in this cohort than in our 2011–2014 cohort (17.15 vs. 13.66%), as was the proportion of eyes with AL > 28 mm (11.28 vs. 9.29%)^14^. The increasing prevalence of high myopia has also been noted in the USA and Israel^22,23^. This suggests worsening myopia within the cataract population, which may have broad public health implications and which underscores the need for more effective intervention and prevention. In light of the growing prevalence of myopia, hospitals and lens manufacturers may wish to focus on intraocular lenses with lower refractive power, IOL manufacturers should consider expanding the diopter range of monofocal, even bifocal, trifocal, and extended depth of focus intraocular lenses to better meet the needs of this growing population. Meanwhile, broadening the diopter range of toric IOLs may help address the needs of highly myopic patients with astigmatism. Finally, a new generation of intraocular lenses that can flexibly address patients’ refractive needs—such as power adjustable IOLs—may also be a promising and necessary development direction not only for eyes with extreme AL, but also eyes with previous corneal refractive surgeries.

Nearly one million corneal refractive surgeries are performed each year in China^24^. Among the patients in our 2020–2023 cohort with a history of refractive surgery, most had undergone either LASIK (46.23%) or photorefractive keratectomy (31.60%), probably reflecting that these are the most widely used types of excimer laser refractive surgery around the world^21,25^. A substantial proportion of patients in our cohort had undergone radial keratotomy, reflecting its popularity in China during the late 1980s and early 1990s^21^. This type of surgery is being phased out because it may be less safe and predictive than other types^20,21,26^, so the prevalence of such surgical history among cataract patients in China should decrease in the future. Few of the patients in our 2020–2023 cohort underwent small-incision lenticule extraction, which was introduced in China around 2006, or implantation of a collamer lens, introduced in China around 2011. Individuals who underwent these types of surgery have not yet reached the age at which cataracts typically develop. We predict that within 1–2 decades, the prevalence of cataract patients with a history of these two types of surgery will be much higher.

In our 2020–2023 cohort as well as our 2010s cohort^14^, the ocular biometric parameters AL, ACD, AQD and WTW tended to decrease, while MK and LT tended to increase with increasing age. These findings are consistent with our previous study and other studies in Bosnia, Israel, and South Korea^14,27–30^. Both ACD and AQD in our 2020–2023 cohort were deeper in eyes that had undergone refractive surgery than in those that had not, likely reflecting the longer AL in the first group^31,32^. Clinicians should bear these differences in mind because AL and ACD are crucial for predicting the effective position of an intraocular lens^33–35^, which can substantially affect refractive outcomes after cataract surgery^36,37^. In our 2020–2023 cohort, the lens was thinner and WTW longer in eyes that had undergone refractive surgery than in those that had not, probably reflecting the younger age and longer AL of the first group^38,39^.

It seems that there was no significant difference in the distribution of astigmatism magnitude between our 2020s and 2010s cohorts. The proportion of eyes with CA < 0.5 D in this cohort was similar to that of our 2011–2014 cohort (28.26 vs. 26.94%), as was the proportion of eyes with CA of 0.5-1 D (36.53 vs. 37.52%), 1–2 D (27.15 vs. 27.03%), 2–3 D (5.98 vs. 5.76%), > 3 D (2.08 vs. 2.75%)^14^. Our analyses of cataract patients in southwest China and a similar analysis of patients in South Korea showed that with increasing age, CA initially decreases then later increases, and that the astigmatism axis shifts from WTR to ATR, with OBL astigmatism being less prevalent than either WTR or ATR, similar to our 2011–2014 cohort^14,30^. As a result, the severity of astigmatism is relatively low among cataract patients 60–69 years old. The severity and axis of astigmatism also depend on previous history of refractive surgery: in our 2020–2023 cohort, the astigmatism amount and the prevalence of different axes in individuals aged 40 to 60 differed between those with a history of refractive surgery and those without. In this study, a higher proportion of patients with a history of refractive surgery had astigmatism greater than 0.75 diopters, which may be attributed to surgically induced changes in corneal curvature or residual/recurrent astigmatism from initially higher preoperative refractive errors. Additionally, refractive surgery may alter the distribution of astigmatic axes due to corneal remodeling, leading to a higher proportion of OBL and ATR astigmatism and a reduced proportion of WTR astigmatism compared to patients without refractive surgery. In fact, the severity of astigmatism may depend on the type of previous surgery: astigmatism was significantly worse among patients who had undergone radial keratotomy than among those who had undergone other types of surgery. Radial keratotomy is known to induce both regular and irregular astigmatism, altering posterior corneal spherical power in unpredictable ways^20,40^. We expect that in the future, clinicians will increasingly turn to toric intraocular lenses^41^ or astigmatic keratotomy, especially femtosecond laser-assisted arcuate keratotomy^42^, to correct astigmatism in cataract patients with a history of refractive surgery.

Our analysis of A/P ratio in the 2020–2023 cohort is consistent with the fact that photorefractive keratectomy and LASIK increase the curvature radius of the anterior corneal surface much more than the curvature radius of the posterior surface, while the opposite is true of radial keratotomy^43^. Clinicians must take into account these effects of previous refractive surgery because they may render intraocular lens calculations inaccurate^44,45^. The Gullstrand paraxial model stipulates a corneal curvature ratio of 7.7 : 6.8 (front : back) by assuming the cornea to be a spherocylinder, yet this assumption is invalid if the eye has undergone corneal refractive surgery^46^. In this case, central corneal power has been proposed to predict the effective lens position, but this approach is prone to errors^6,44^. Given that an increasing proportion of cataract patients will come to the clinic with high myopia and/or a history of refractive surgery, new methods to calculate intraocular lens power are urgently needed that can accurately handle these challenging cases. Artificial intelligence may be useful in meeting these demands.

This study examined a relatively large sample of cataract patients during four years, which is sufficiently long to observe trends and reduce noise due to natural but unpredictable fluctuations. While our sample came from a large geographic area in southwest China, it may not be entirely representative of patient populations elsewhere in China or in the world. Nevertheless, our sample came from the same medical center as our previous analysis a decade ago, allowing a pseudo-longitudinal assessment of the changing demographic and ocular biometric characteristics of cataract patients in southwest China. Since the age inclusion criteria differed between the two cohorts (≥ 40 years in the 2020s cohort vs. ≥50 years in the 2010s cohort), the comparisons should be performed and interpreted with caution.

Our study of the large cohort of cataract patients in southwest China in early 2020s indicates that cataract patients today are younger and more likely to have high myopia and a history of corneal refractive surgery than a decade ago. Clinicians and designers of intraocular lenses should take into account the changing demographic and ocular biometric characteristics of cataract patients in order to optimize surgical outcomes, which will require more accurate and robust methods to calculate intraocular lens power as well as potentially better lenses to correct high myopia and astigmatism.

## Methods

Medical records were retrospectively reviewed for a consecutive series of individuals at least 40 years old who underwent cataract surgery at West China Hospital of Sichuan University (Chengdu, China) between January 2020 and December 2023. The study protocol was approved by the Ethics Committee of West China Hospital (2024–963). Due to the retrospective nature of the study, the Ethics Committee of West China Hospital waived the need of obtaining informed consent because patients or their legal guardians, at the time of surgery, consented to the analysis and publication of anonymized medical data for research purposes. The study was carried out in accordance with the Declaration of Helsinki.

**Fig. 5:**
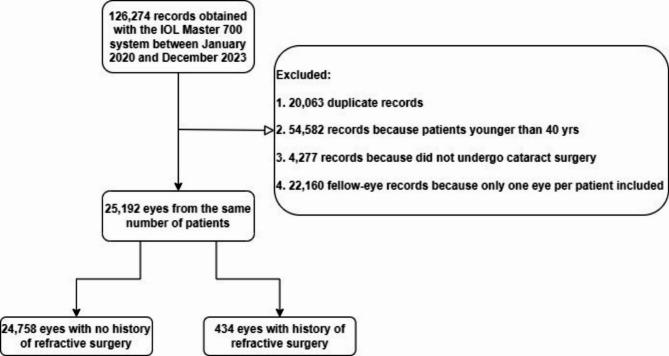
Flowchart of patient selection and stratification.

Ocular biometric parameters were determined using the IOL Master 700 (Carl Zeiss Meditec, Jena, Germany). The following data were extracted for each subject (Fig. 5): age, sex, previous refractive surgery, keratometry (K, calculated as the average of minimal and maximal keratometry), axial length (AL), central corneal thickness (CCT), anterior chamber depth (ACD), aqueous depth (AQD), lens thickness (LT) and horizontal corneal diameter (“white-to-white”, WTW). AL, ACD, AQD, LT, CCT were measured at least five times, and they were calculated by averaging all measurements. Corneal astigmatism (CA) was calculated as the absolute difference between the minimal and maximal corneal radii, and it was classified as “with-the-rule” (WTR) when the steep meridian on the corneal surface was 60–120 degrees, “against-the-rule” (ATR) when the steep meridian was 0–30 or 150–180 degrees, or “oblique” (OBL) in other cases. Mean total keratometry (MTK), mean anterior keratometry (MAK), and mean posterior keratometry (MPK) were calculated by averaging the corresponding minimal and maximal radius. A/P was defined to be the ratio of anterior to posterior corneal surface (A/P). We analyzed only one eye from each patient. In patients who underwent cataract surgery in both eyes, we selected the right eye for analysis because ocular biometric characteristics are similar within pairs of eyes^47^. If patients had a history of refractive surgery in only one eye, that eye was selected for analysis.

Statistical analyses were performed using Microsoft Excel (Microsoft, Redmond, WA, USA) and GraphPad Prism 8.3.0 (GraphPad Software, San Diego, CA, USA). Continuous data were reported as mean ± standard deviation, while categorical data were reported as n (%). Frequency distributions of ocular biometric parameters were fitted using a nonlinear Gaussian model. In some comparisons, cataract surgery patients were stratified according to whether they had a history of refractive surgery and further stratified according to the type of refractive surgery. Differences in categorical variables were assessed for significance using the chi-squared test. Continuous variables between two groups were compared using an unpaired t-test with Welch’s correction. Differences among three or more groups were assessed using Tukey’s multiple-comparisons tests and Kruskal-Wallis tests. Differences were considered significant if associated with *P* < 0.05.

## Electronic supplementary material

Below is the link to the electronic supplementary material.

Supplementary Material 1

## Data Availability

The raw data supporting the conclusions of this article will be made available by J.M. (nkmujiancheng@163.com), without undue reservation.
