# Supplementary material for: Updated study on demographic and ocular biometric characteristics of cataract patients indicates new trends in cataract surgery
Source: Sci Rep. 2025 May 19;15:17289. doi: 10.1038/s41598-025-02311-5 (PMC12089336; doi:10.1038/s41598-025-02311-5)

Supplemental Online Content

Supplementary table 1. Demographic and ocular biometric characteristics of our cohort, stratified by age group

Supplementary table 2. Frequencies of different types of refractive surgery in our cohort, stratified by year

Supplementary table 3. Prevalences of different ranges of axial length, mean K and corneal astigmatism in the entire cohort or in subgroups stratified by history of refractive surgery

Supplementary table 4. Prevalences of different ranges of axial length across age groups in the cohort

Supplementary figure 1. Distribution of ocular biometric characteristics in the entire cohort

Supplementary figure 2. Differences across age groups of the entire cohort in (A) axial length, (B) mean simulated keratometry, (C) corneal astigmatism, (D) anterior chamber depth, (E) aqueous depth, (F) lens thickness, (G) mean total keratometry, (H) mean anterior keratometry, (I) mean posterior keratometry, (J) the ratio of anterior to posterior corneal curvature radii (A/P), (K) central corneal thickness, and (L) horizontal corneal diameter ("white-to-white")

Supplementary figure 3. Comparison of the distributions of ocular parameters between patients with a history of refractive surgery (RS, blue) and those without such history (NRS, green)

Supplementary figure 4. Comparison of ocular biometric parameters among subgroups of patients in the cohort with no history of refractive surgery (NRS) or a history of radial keratotomy (RK), photorefractive keratectomy (PRK) or laser in-situ keratomileusis (LASIK)

Supplementary table 1. Demographic and ocular biometric characteristics of our cohort, stratified by age group

Characteristic	Age, yr					Total	*P*
	40-49	50-59	60-69	70-79	>80		
n (% of 25,192)	2,728 (10.83)	6,839 (27.15)	7,476 (29.68)	5,960 (23.66)	2,189 (8.69)	25,192 (100)	
Age, yr	45.28±2.94	54.84±2.83	64.97±2.80	73.99±2.82	83.56±3.34	63.84±11.23	<0.0001
Sex							
Men	1,245 (45.64)	2,965 (43.35)	2,897 (38.75)	2,405 (40.35)	1,058 (48.33)	10,570 (41.96)	
Women	1,483 (54.36)	3,874 (56.65)	4,579 (61.25)	3,555 (59.65)	1,131 (51.67)	14,622 (58.04)	
AL (mm)	25.68±3.14	25.26±3.05	24.08±2.33	23.88±1.92	23.74±1.48	24.50±2.60	<0.0001
MK (D)	43.44±2.32	43.70±2.06	44.11±1.65	44.22±1.56	44.18±1.56	43.96±1.85	<0.0001
CA (D)	1.05±0.90	0.96±0.93	0.83±0.80	0.95±0.79	1.11±0.86	0.95±0.85	<0.0001
ACD (mm)	3.27±0.46	3.17±0.48	2.98±0.48	2.92±0.49	2.86±0.46	3.04±0.50	<0.0001
AQD (mm)	2.73±0.46	2.63±0.49	2.44±0.49	2.38±0.49	2.33±0.46	2.50±0.50	<0.0001
LT (mm)	4.20±0.44	4.34±0.50	4.48±0.50	4.59±0.51	4.69±0.52	4.45±0.52	<0.0001
MTK (D)	43.42±2.35	43.62±2.15	44.10±1.65	44.23±1.56	44.26±1.51	43.94±1.88	<0.0001
MAK (D)	48.39±2.59	48.68±2.30	49.14±1.84	49.26±1.74	49.22±1.74	48.97±2.06	<0.0001
MPK (D)	-5.89±0.29	-5.90±0.28	-5.93±0.25	-5.93±0.24	-5.90±0.23	-5.92±0.26	<0.0001
A/P	1.14±0.04	1.14±0.04	1.13±0.02	1.13±0.02	1.12±0.02	1.14±0.03	<0.0001
CCT (mm)	0.5399±0.0457	0.5397±0.0420	0.5381±0.0389	0.5376±0.0405	0.5369±0.0411	0.5385±0.0411	<0.01
WTW (mm)	11.83±0.49	11.74±0.45	11.62±0.44	11.56±0.45	11.58±0.45	11.66±0.46	<0.0001

Values are n (%) or mean ± SD. ACD: anterior chamber depth; AL: axial length; A/P: ratio of anterior to posterior corneal surface; AQD: aqueous depth; CA: corneal astigmatism; CCT: central corneal thickness; LT: lens thickness; MAK: mean anterior keratometry; MK: mean simulated keratometry; MPK: mean posterior keratometry; MTK: mean total keratometry; WTW: white-to-white.

Supplementary table 2. Frequencies of different types of refractive surgery in our cohort, stratified by year

Surgery type	Year				Total
	2020	2021	2022	2023	
Radial keratotomy	7 (14.89)	13 (14.13)	23 (18.70)	38 (23.60)	81 (19.10)
Photorefractive keratectomy	14 (29.79)	30 (32.61)	34 (27.64)	56 (34.57)	134 (31.60)
Laser-assisted sub-epithelial keratectomy	1 (2.13)	1 (1.09)	2 (1.63)	6 (3.70)	10 (2.36)
Laser in-situ keratomileusis	25 (53.19)	46 (50.00)	64 (52.03)	61 (37.65)	196 (46.23)
Implantation of collamer lens	0 (0)	2 (2.17)	0 (0)	1 (0.62)	3 (0.71)
All types	47 (11.08)	92 (21.70)	123 (29.01)	162 (38.21)	424 (100)

Values are n (%).

Supplementary table 3. Prevalences of different ranges of axial length, mean K and corneal astigmatism in the entire cohort or in subgroups stratified by history of refractive surgery

Parameter	Previous surgery	No previous surgery	Total
n (% of 25,192)	434 (1.72)	24,758 (98.28)	25,192 (100)
Axial length (mm)			
< 22	0 (0)	1457 (5.88)	1457 (5.78)
22-24	5 (1.15)	13273 (53.61)	13278 (52.71)
24-26	61 (14.06)	5442 (21.98)	5503 (21.84)
26-28	131 (30.18)	1981 (8.00)	2112 (8.38)
28-30	108 (24.88)	1281 (5.17)	1389 (5.51)
30-32	90 (20.74)	798 (3.22)	888 (3.52)
> 32	39 (8.99)	526 (2.12)	565 (2.24)
Mean K (D)			
< 36	97 (22.35)	25 (0.10)	122 (0.48)
36-38	131 (30.18)	27 (0.11)	158 (0.63)
38-40	130 (29.95)	118 (0.48)	248 (0.98)
40-42	60 (13.82)	2057 (8.31)	2117 (8.40)
42-44	14 (3.23)	9723 (39.27)	9737 (38.65)
44-46	2 (0.46)	10185 (41.14)	10187 (40.44)
> 46	0 (0)	2623 (10.59)	2623 (10.41)
Corneal astigmatism (D)			
< 0.25	28 (6.45)	2224 (8.98)	2252 (8.94)
0.25-0.5	63 (14.52)	4805 (19.41)	4868 (19.32)
0.5-0.75	82 (18.89)	5020 (20.28)	5102 (20.25)
0.75-1.0	76 (17.51)	4025 (16.26)	4101 (16.28)
1.0-1.5	113 (26.04)	4674 (18.88)	4787 (19.00)
1.5-2.0	42 (9.68)	2010 (8.12)	2052 (8.15)
2.0-2.5	14 (3.23)	837 (3.38)	851 (3.38)
2.5-3.0	8 (1.84)	647 (2.61)	655 (2.60)
> 3.0	8 (1.84)	516 (2.08)	524 (2.08)

Values are n (%).

Supplementary table 4. Prevalences of different ranges of axial length across age groups in the cohort

	Age, yr				
Axial length (mm)	40-49	50-59	60-69	70-79	≥80
< 22	137 (5.02)	377 (5.51)	510 (6.82)	340 (5.70)	93 (4.25)
22-24	868 (31.82)	2786 (40.74)	4487 (60.02)	3729 (62.57)	1408 (64.32)
24-26	680 (24.93)	1510 (22.08)	1455 (19.46)	1300 (21.81)	558 (25.49)
26-28	471 (17.27)	891 (13.03)	410 (5.48)	271 (4.55)	69 (3.15)
28-30	256 (9.38)	607 (8.88)	306 (4.09)	177 (2.97)	43 (1.96)
30-32	190 (6.96)	388 (5.67)	190 (2.54)	106 (1.78)	14 (0.64)
> 32	126 (4.62)	280 (4.09)	118 (1.58)	37 (0.62)	4 (0.18)

Values are n (%).

Supplementary figure 1. Distribution of ocular biometric characteristics in the entire cohort. (A) Axial length. (B) Mean simulated keratometry. (C) Corneal astigmatism. (D) Anterior chamber depth. (E) Aqueous depth. (F) Lens thickness. (G) Mean total keratometry. (H) Mean anterior keratometry. (I) Mean posterior keratometry. (J) Central corneal thickness. (K) Horizontal corneal diameter ("white-to-white").

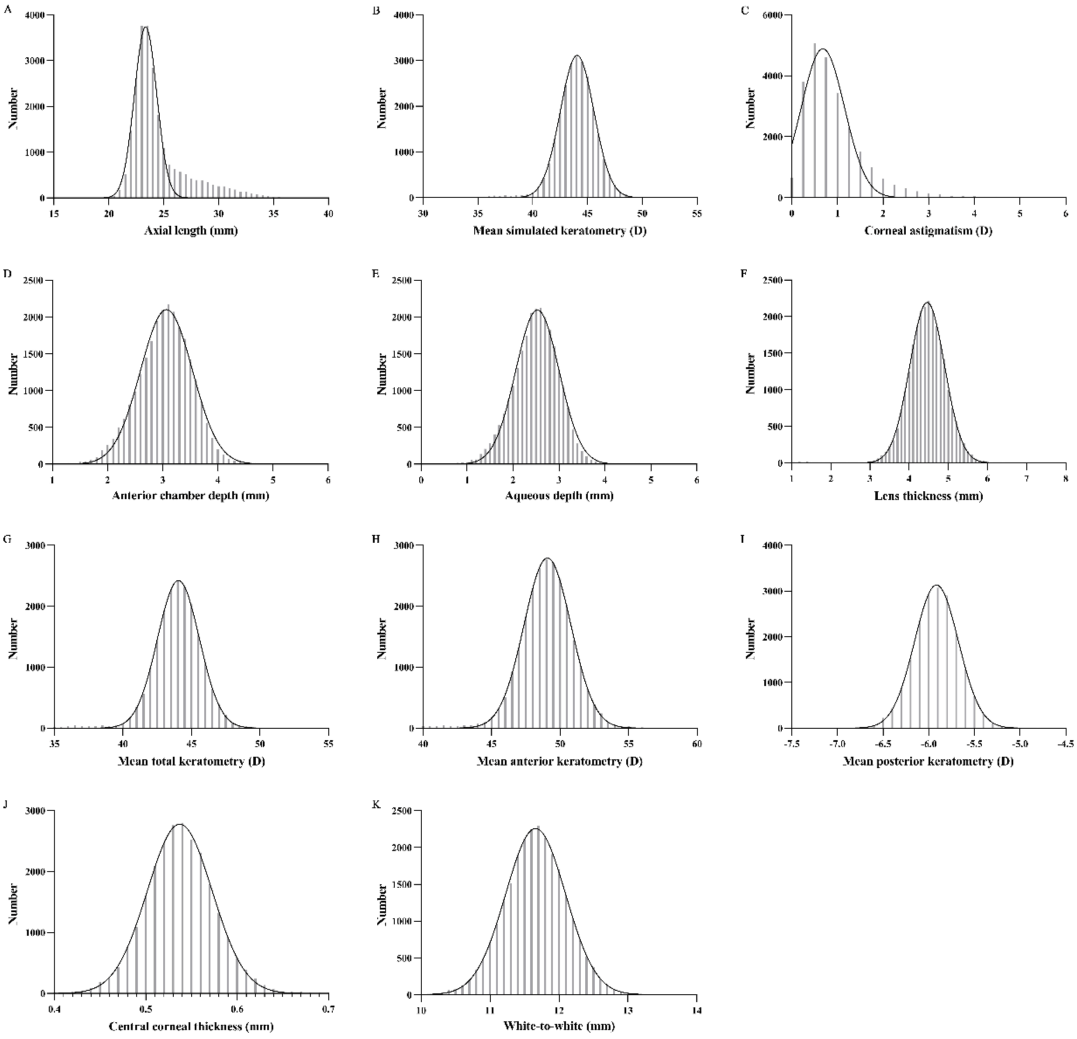

Supplementary figure 2. Differences across age groups of the entire cohort in (A) axial length, (B) mean simulated keratometry, (C) corneal astigmatism, (D) anterior chamber depth, (E) aqueous depth, (F) lens thickness, (G) mean total keratometry, (H) mean anterior keratometry, (I) mean posterior keratometry, (J) the ratio of anterior to posterior corneal curvature radii (A/P), (K) central corneal thickness, and (L) horizontal corneal diameter ("white-to-white"). **$p < 0.01$, ***$p < 0.001$, ****$p < 0.0001$.

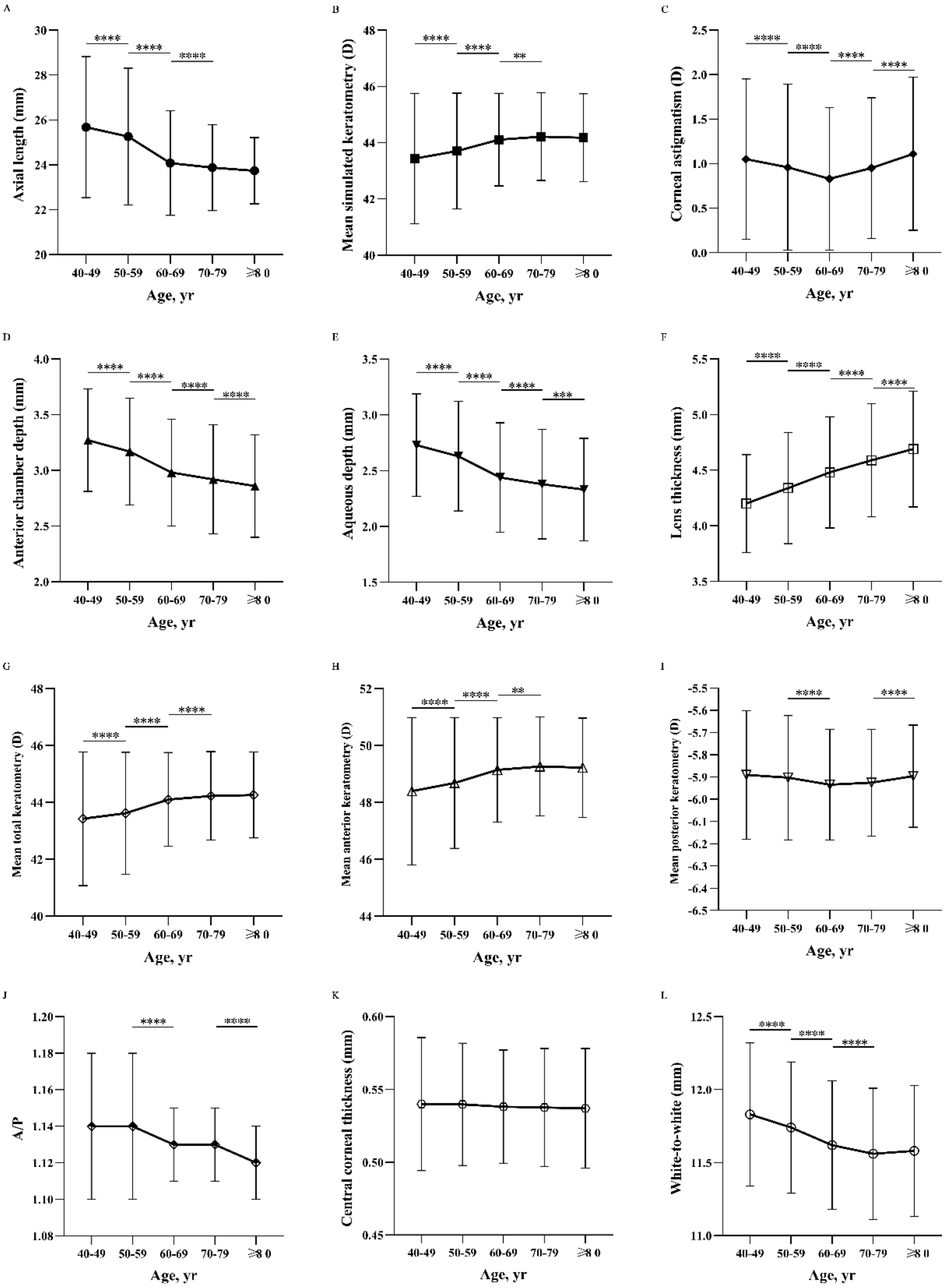

Supplementary figure 3. Comparison of the distributions of ocular parameters between patients with a history of refractive surgery (RS, blue) and those without such history (NRS, green). (A, axial length; B, mean simulated keratometry; C, corneal astigmatism; D, anterior chamber depth; E, aqueous depth; F, lens thickness; G, mean total keratometry; H, mean anterior keratometry; I, mean posterior keratometry; J, central corneal thickness; K, white-to-white)

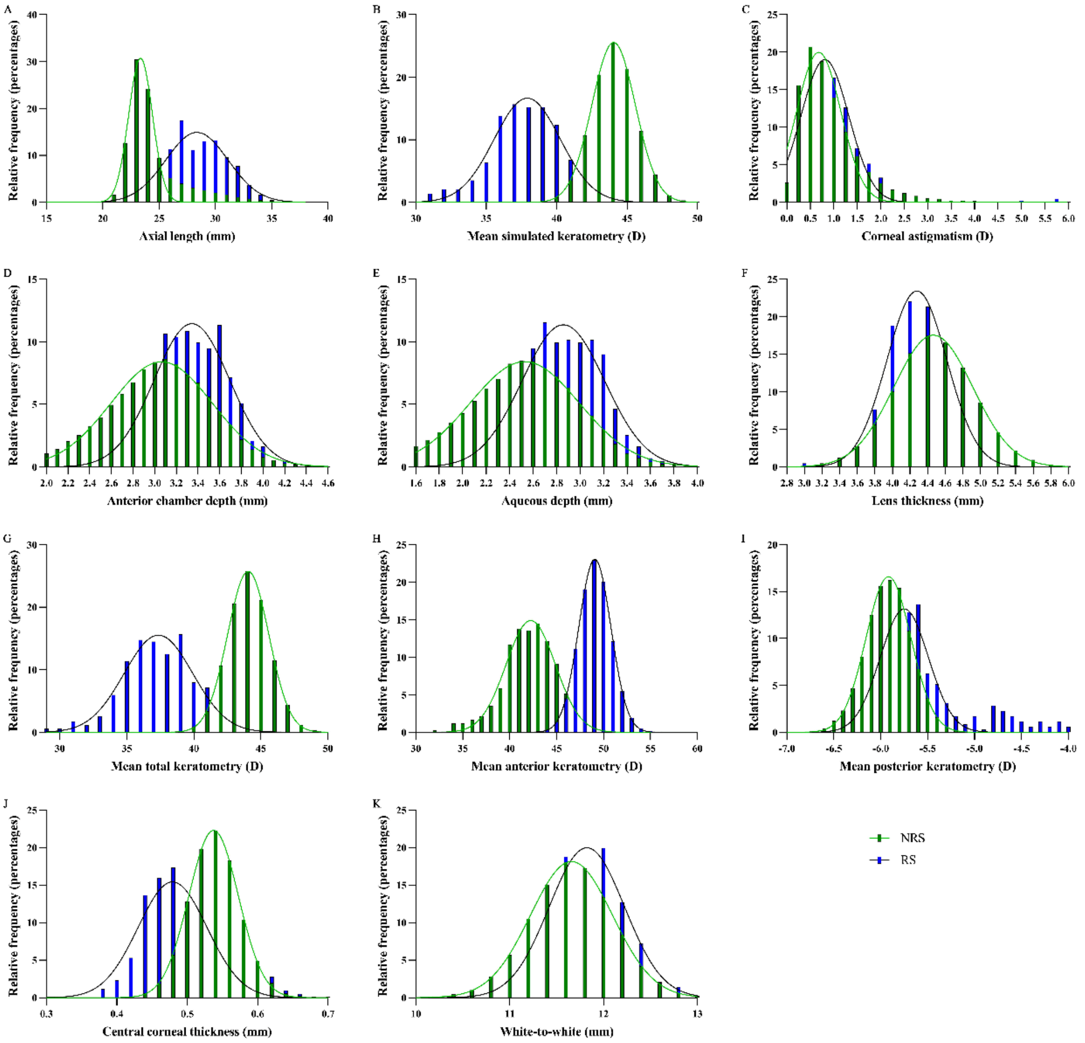

Supplementary figure 4. Comparison of ocular biometric parameters among subgroups of patients in the cohort with no history of refractive surgery (NRS) or a history of radial keratotomy (RK), photorefractive keratectomy (PRK) or laser in-situ keratomileusis (LASIK). (A, axial length; B, mean simulated keratometry; C, corneal astigmatism; D, anterior chamber depth; E, aqueous depth; F, lens thickness; G, mean total keratometry; H, mean anterior keratometry; I, mean posterior keratometry; J, the ratio of anterior to posterior corneal curvature radii (A/P); K, central corneal thickness; L, white-to-white) **p* < 0.05, ***p* < 0.01, ****p* < 0.001, *****p* < 0.0001.

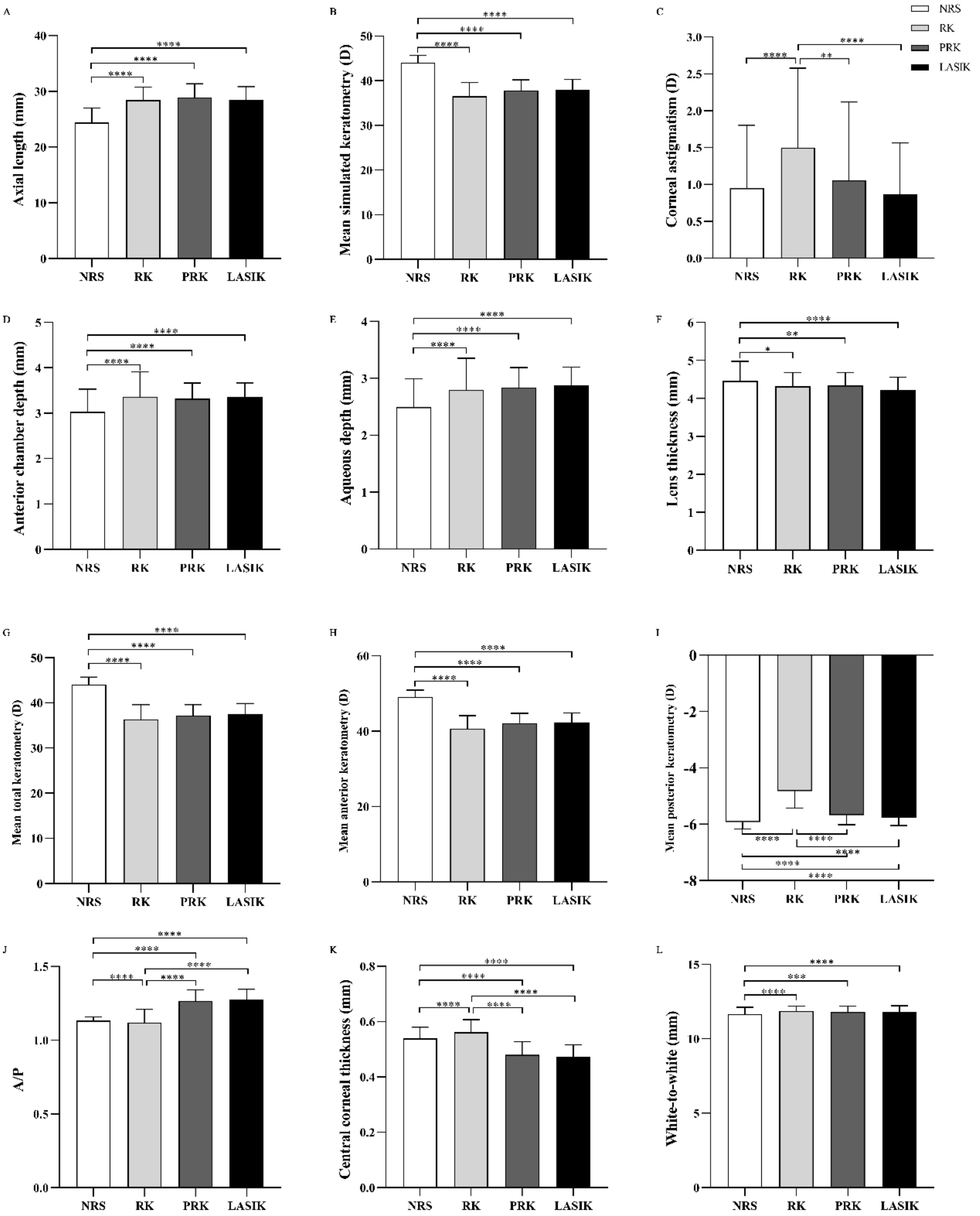

Supplement: Supplementary file 1 — Supplementary Material 1 [file 41598_2025_2311_MOESM1_ESM.pdf]
